# Whole exome sequencing of lung adenocarcinoma and lung squamous cell carcinoma in one individual: A case report

**DOI:** 10.1111/1759-7714.13540

**Published:** 2020-06-23

**Authors:** Yuxi Bai, Yinghui Xu, Xu Wang, Yunpeng Liu, Chao Sun, Ye Guo, Yangyang Cai, Guoguang Shao, Zhiguang Yang, Shi Qiu, Kewei Ma

**Affiliations:** ^1^ Cancer Center The First Hospital of Jilin University Jilin China; ^2^ Thoracic Surgery Department The First Hospital of Jilin University Jilin China

**Keywords:** Multiple primary lung cancers (MPLCs), non‐small cell lung cancer (NSCLC), whole exome sequencing (WES)

## Abstract

Multiple primary lung cancers (MPLCs) refers to two or more primary malignant tumors that occur simultaneously or successively in the lung of the same patient. Distinguishing MPLCs from intrapulmonary metastases is important for treatment strategy and prognosis. MPLCs have been considered as having different origins and clonal evolutionary processes. Whole genome sequencing (WGS) and whole exome sequencing (WES) are regarded as an effective way to identify the relationship and differentiation among MPLC nodules. Here, we report the case of a 63‐year‐old MPLC male patient who smoked for 40 years. Two nodules were found on chest computed tomography (CT) scan, which were further confirmed by pathology to be lung adenocarcinoma (ADC) and lung squamous cell carcinoma (SCC), respectively. WES of the two different nodules was performed, and the results showed that there was a significant genetic difference between the two nodules. Further analysis of the tumor mutation burden (TMB) of the two tumor lesions showed that the TMB of the squamous cell carcinoma was higher than that of the adenocarcinoma, indicating that the squamous cell carcinoma had a higher mutation frequency. According to the pathology and WES sequencing results, MPLCs for this case were regarded as independent of each other, with different origins and clonal evolutionary processes. In this case report, we emphasize that WES should play an important role in determining the origin of MPLC clones, and also make some explorations for the further discovery of new potential driver genes and therapeutic targets.

## Graphical abstract

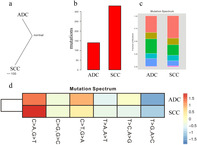

## Introduction

Multiple primary lung cancers (MPLCs) refers to two or more primary malignant tumors occurring simultaneously or successively in the lung of the same patient.^1^, ^2^ The incidence of MPLC has been reported to be 0.2%–8%.^3^, ^4^, ^5^ Some studies have reported that MPLCs are independent, with different origins and different clonal evolutionary processes.^6^ To explore the relationship and differentiation among these MPLCs, multiple cancer research centers have conducted systematic and comprehensive analyses of MPLCs at the molecular level.^7^ Next‐generation sequencing (NGS) is one of the most valuable molecular diagnostic techniques in recent years. NGS has been widely used in the detection of hereditary germline mutations and acquired somatic mutations. Whole genome sequencing (WGS) and whole exome sequencing (WES) can screen the entire genome‐encoded exome spectrum and identify single nucleotide variants (SNVs), which can help us understand and distinguish each MPLC lesion genetically. Our team has been working on MPLCs for years. We have collected many cases of MPLCs, all of which have been operated on after diagnosis. Surgical specimens were used after obtaining the patient's permission, and WES was performed of the MPLC samples of each patient. This article reports a case of MPLCs. The pathologic results of the two lung nodules suggested that they were completely different; one was lung adenocarcinoma (ADC), and the other was lung squamous cell carcinoma (SCC). To address this, we performed WES of the two nodules and analyzed the genetic differences between the two lesions.

## Case report

**Figure 1:**
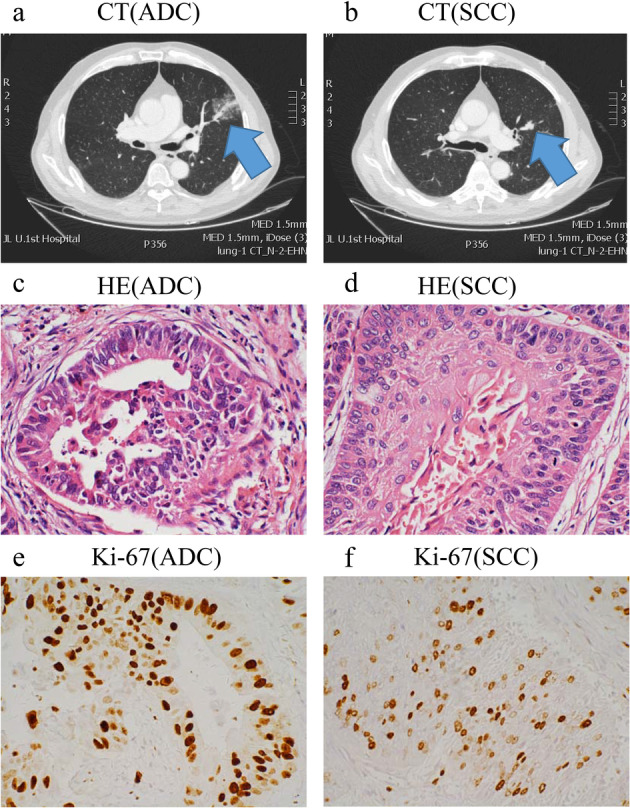
Preoperative lung computed tomography (CT) scans of this patient. (**a**) CT image of adenocarcinoma; and (**b**) CT image of squamous cell carcinoma. (**c–f**) Hematoxylin and eosin stained images of ADC (**c**), SCC (**d**) (original magnification 40x). Immunohistochemistry of ADC (**e**) and SCC (**f**) reported positive staining for Ki‐67 (original magnification 40x).

The patient was a 63‐year‐old male Asian who had smoked for more than 40 years, averaging approximately 400 packs per year. He was admitted to the hospital after examination revealed two nodules located in the left upper lobe of his lung. One nodule was about 1.5 cm (Fig 1a), the other was 1.0 cm (Fig 1b). Following completion of preoperative preparation, the patient underwent left upper lobe lobectomy and lymphadenectomy. Two nodules were found in the resected lobes postoperatively by the pathology department, one of which was pathologically confirmed as invasive adenocarcinoma in the left upper lobe (volume: 1.7 × 1.5 × 1.3 cm) (Fig 1c) and infiltrating keratotic squamous cell carcinoma differentiated in the left upper lobe (diameter: approximately 1.0 cm) (Fig 1d). The immunohistochemistry results were as follows: left upper lobe adenocarcinoma: Ki‐67 (+ 30%) (Fig 1e), (a few +), vera.ttf ‐ 1 CK7 (+), NapsinA (−), WT ‐ 1 Villin (+), CK20 (−), ventana: contrast ALK (−), lung squamous cell carcinoma: ki‐67 (+20%) (Fig 1f), ttf‐1 (−), ck5/6 (+), P40 (+), and CK7 (−).

WES of the two lung nodules was performed using the Illumina NovaSeq sequencer to test the relationship and differences from the genetic level among the two lesions.

**Figure 2:**
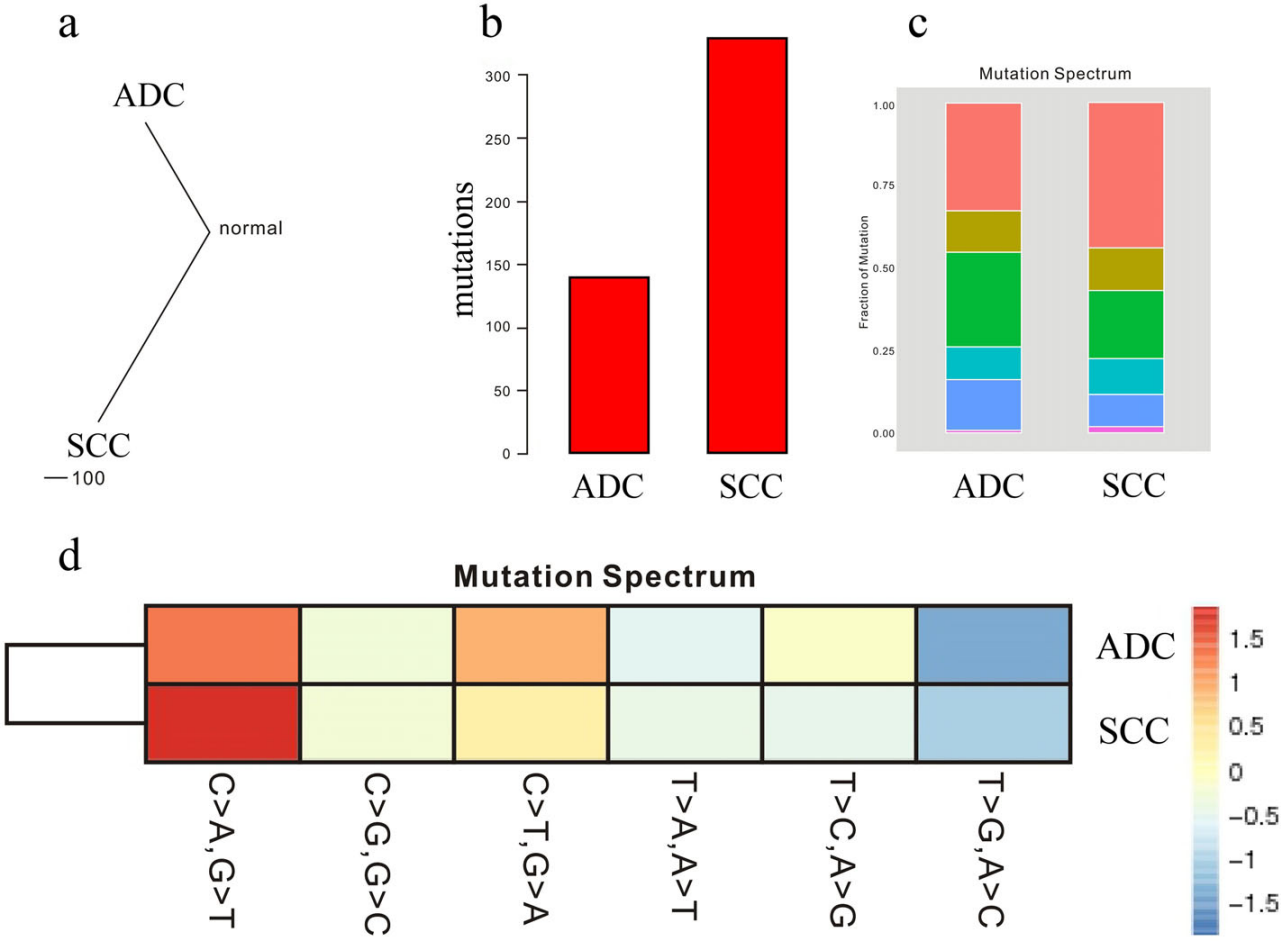
(**a**) Shows the dispersion analysis of the two tumors in this patient. (**b**) According to somatic cell mutation data, we counted the number of nonsynonymous mutations in credible somatic cell mutations to obtain the tumor mutation burden (TMB) of tumor tissues. (**c**) Mutation spectrum 1. The x‐coordinate represents the different tumors, and the ordinate is the ratio of different types of mutant bases. This figure shows the different proportions of different types of mutant bases (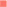) C>A, G>T, (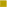) C>G, G>C, (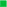) C>T, G>A, (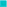) T>A, A>T, (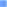) T>C, A>G, and (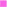) T>G, A>C. (**d**) Mutation spectrum 2. This shows a mutation spectrum where the different proportions of different types of mutations in lung SCC and ADC are expressed by different colors. The x‐coordinate shows the six mutation types. The ordinate represents the different tumors. The more red the heat map grid color, the greater the proportion of this type of mutation in the corresponding tumor. The more blue the grid color, the smaller the proportion of this type of mutation in the corresponding tumor.

We also performed a dispersion analysis on the two nodules of this patient (Fig 2a) where it could be seen that the two nodules had a dispersion of 100, which meant that they were completely different. The similarity of the two tumors was zero, and they were independent of each other.

We used Illumina NovaSeq sequencer to complete the whole exome sequencing (WES) of the lung tumors and used 200PE. The sequencing depth of the two lung tumors was 150X. The white blood cell genome was the control sample, and its sequencing depth was 100X. We used GATK depth of coverage software to calculate the genome sequencing depth of each tumor and used MuTect2 (GATK V3.6) software to analyze somatic mutations. Credible somatic cell mutation data were screened based on information, such as mutation depth, and the number of nonsynonymous mutations in the credible somatic cell mutations was counted to obtain the TMB of tumor tissues. Lung adenocarcinoma showed 136 mutation sites, and lung squamous cell carcinoma showed 335 mutation sites (Fig 2b). The TMB of lung squamous cell carcinoma was also greater than that of lung adenocarcinoma.

Genetic single nucleotide polymorphism (SNPs) were analyzed with Unified Genotyper, Haplotype Caller and atlas2 software, and insert‐deletion mutations (Indel) of heritability were analyzed with atlas and platypus software. Annovar software was used to annotate the detected gene mutations and complete the detection of hereditary gene mutations. We identified 134 single nucleotide variants (SNVs) in lung ADC and 325 SNVs in lung SCC, as well as insertion‐deletion (InDel), structural variation (SV) and copy number variation (CNV) (Fig 2c,d). According to the SNV detection results of the two samples, we calculated the ratio of all the replacement types of six single bases, C > A/G > T, C > G/G > C, C > T/G > A, T > A/A > T, T > C/A > G, T > G/A > C. In lung ADC, the dominant one is the substitution of C > A/G > T followed by the substitution of C > T/G > A. Lung SCC is also dominated by the substitution of C > A/G > T followed by the substitution of C > T/G > A. The variation of the two samples was similar.

## Discussion

In this report, we describe an infrequent case of a patient who had two histologically distinct intrapulmonary tumors. We performed WES of the two tumors to establish a clonal relationship. The two different lung tumors of this patient presented different pathological and molecular states, despite having the same genetic background and internal environment. This finding indicates that each tumor in this individual had its own origin and clonal evolution and eventually formed different phenotypes.

With the application of immune checkpoint inhibitors (ICIs) in clinical practice in recent years, the concept of TMB has gradually become a hot research topic in the oncology field. TMB is a new biomarker for predicting the response to programmed cell death ligand‐1 (PD‐L1) treatment. TMB is commonly defined as the total number of somatic coding mutations and is associated with the emergence of neoantigens that trigger antitumor immunity. Common detection techniques include WGS, WES, and gene‐targeted sequencing. Furthermore, the lung SCC gene has been reported to have a higher TMB or mutation burden (MB) than adenocarcinomas.^8^ It has been hypothesized that tumors with a higher MB are more likely to express neoantigens and to induce a more robust immune response in the presence of ICIs. Another important consideration is that lung SCC may benefit more from treatment with ICIs. For the two lung tumors in our patient, the lung ADC showed 136 mutation sites (Fig 2b), and the lung SCC showed 335 mutation sites (Fig 2b). The TMB of lung SCC was significantly higher than that of lung ADC. The results of this study are consistent with previous reports in the literature. Lung SCC has more gene mutation sites, a higher tumor mutation burden, but there are no targeted drugs available. However, higher TMB is the main reason to promote the generation of neoantigens in the body,^8^ and it is also the main reason why lung SCC may benefit more from treatment with ICIs.^9^, ^10^

For this patient, we know from the dispersion analysis that the dispersion of the two lung tumors was 100 (Fig 2a), which indicates that there was no similarity between them and that they were two independent lung tumors. According to the SNV test results of all the samples, the proportion of replacement types of six single bases was calculated and counted, and the mutation spectrum (Fig 2c,d) of base replacement and the clustering heat map of mutation spectrum (Fig 2d) were obtained. Somatic mutation is induced by mismatches in the process of DNA replication, induction of endogenous or exogenous mutagens and defects in the DNA repair mechanism. Specific combinations of mutation types are generated by different mutation processes. Gene point mutations can be divided into 96 types by considering the base types at 1 bp above and 1 bp below the point mutation site. The display of these 96 types results in a proportional distribution diagram of mutation types, also known as the mutation pattern diagram. Lung ADC and lung SCC showed similar mutation characteristics, which were replaced by C > A/G > T followed by C > T/G > A. It has previously been reported in the literature that in lung ADC and SCC, lung small cell carcinoma, head and neck squamous cell carcinoma, and liver cancer, C > A/G > T is associated with smoking.^11^ In combination with the patient's smoking history, this base substitution is consistent with previous studies.^11^, ^12^, ^13^ The mutation could be due to a combination of cancer‐causing chemicals in cigarettes, which may interact with DNA and lead to the accumulation of somatic mutations. Second, C > T/G > A is positively correlated with age in most childhood and adult cancer types. The patient in this study was a 63‐year‐old elderly patient, and the base changes were consistent with those described in previous reports.^13^, ^14^ The C > T transformation mainly occurs on the NpCpG trinucleotide, and this mutation process plays a role in the germ line, leading to the large consumption of NpCpG sequences and normal somatic cells. This finding may be observed because most of the mutations of C > T occur continuously and stably throughout the life cycle of cancer patients, while other mutations show different degrees of mutation after exposure to different carcinogens.^14^ Therefore, we believe that C > T/G > A mutations are positively correlated with age.

In summary, this study is based on WES of lung ADC and lung SCC in the same patient, and our aim was to analyze the origin and evolution of multiple primary micronodular lung cancer. The intratumor heterogeneity of lung ADC and lung SCC, the genetic susceptibility to cancer, and the search for and identification of more driving genes should be explored in future research. However, more case samples are needed to verify the findings in this study.

## Disclosure

The authors declare that there are no conflicts of interest.
